# Smartphone apps launched for atrial fibrillation patients and their healthcare providers

**Published:** 2018

Novel smartphone and tablet applications (apps) for atrial fibrillation patients and healthcare professionals have been launched by heart experts. The objectives and design of the apps are outlined in an article published online recently in *EP Europace*, with a summary published in the *European Heart Journal*.

Atrial fibrillation is the most common heart rhythm disorder and significantly increases the risk of stroke and death. One in four middle–aged adults in Europe and the US will develop atrial fibrillation, and the incidence and prevalence are rising.

‘Around two–thirds of people in Europe and the US have a mobile device and use it as their main way of accessing online information,’ said lead author Dr Dipak Kotecha, a clinician scientist in cardiovascular medicine at the Institute of Cardiovascular Sciences, University of Birmingham, UK. ‘This presents a big opportunity to improve self–management and shared decision making in atrial fibrillation.’.

The My AF app and AF Manager app were designed by the European Society of Cardiology (ESC) Guidelines Task Force on Atrial Fibrillation and the CATCH ME consortium, of which the ESC is a partner. The apps were developed over the last two years in tandem with the writing of the 2016 ESC guidelines on atrial fibrillation. Both apps are freely available for Android and iOS devices through the Google Play, and Apple stores.

My AF is for patients with atrial fibrillation. It provides information about the condition, the risk of stroke, atrial fibrillation treatments, and tips on improving lifestyle. Patients can record symptoms and quality of life in a diary which can be shared with a nominated health professional before each consultation to maximise face–to–face time.

Developed in collaboration with patients and patient support groups, My AF provides high–quality information in a simple format that is suitable for adults of all ages. Work is underway to translate the app into several languages.

Dr Kotecha said: ‘The app aims to encourage active patient involvement in the management of their condition. There is evidence that patient education can improve selfcare, adherence to therapy, and long–term outcomes.’

AF Manager is for doctors, nurses and other healthcare professionals. It is the first app of its kind to be submitted for CE certification and is in the final stages of approval. AF Manager imports information shared by the patient and allows the healthcare professional to amend details and enter other medical information, such as electrocardiogram or echocardiography data. The Treatment Manager tool within the app then suggests individualised treatment options based on ESC guidelines. After a consultation, the notes, treatment decisions and medication dosages can be entered and then shared with the patient.

‘Many studies have shown that when clinicians follow guideline recommendations, patients have better outcomes,’&quot; said Dr Kotecha. ‘All of the decision aids in AF Manager are based on ESC guidelines so we hope this will encourage guideline implementation. Patients will have the option to anonymously donate their data, which will enable us to assess the guideline adherence rate.’

The apps are linked to allow transfer of data between patients and healthcare professionals via a secure server at the University of Birmingham, UK. Patients control who can view and edit their data. When data sharing is enabled, updates are synced on both apps. All shared information is encrypted and password protected.

Dr Kotecha said: ‘We know that effective management of atrial fibrillation is suited to shared decision making and we have created the apps in the hope of facilitating this process. Sharing information should save clinicians time and enable them to devote consultations to choosing the best treatments.’

He concluded: ‘The dynamic nature of this technology will allow us to modify and update the features and content to reflect feedback from users, as well as future versions of the ESC atrial fibrillation guidelines.’

## References

[R01] European Society of Cardiology Press Office.

